# Pull-Down Assay-Guided Insights into the Effects of Latroeggtoxin-VI on Nerve Cells

**DOI:** 10.3390/toxins13020136

**Published:** 2021-02-12

**Authors:** Xiaochao Tang, Dianmei Yu, Haiyan Wang, Wenwen Meng, Yiwen Zhai, Zhixiang Lei, Zhen Liu, Xianchun Wang

**Affiliations:** State Key Laboratory of Developmental Biology of Freshwater Fish, Protein Chemistry Laboratory, College of Life Sciences, Hunan Normal University, Changsha 410081, China; Tangxiaochao92@163.com (X.T.); Yudianmei1@126.com (D.Y.); Wanghaiyan@hunnu.edu.cn (H.W.); Meng_wnewnen@126.com (W.M.); Zhaiyiwen1069@126.com (Y.Z.); Leizhixiang202102@163.com (Z.L.); Liuzhen0614@163.com (Z.L.)

**Keywords:** Latroeggtoxin-VI, interaction protein, pull-down, mass spectrometry, PC12 cell, *L. tredecimguttatus* egg

## Abstract

Latroeggtoxin-VI (LETX-VI) is a peptide neurotoxin newly found from the eggs of spider *L. tredecimguttatus.* To explore the mechanism of action of the LETX-VI on nerve cells, the effects of LETX-VI on PC12 cells, a commonly used neuron model, were analyzed using a pull-down assay-guided strategy. LETX-VI was shown to interact with 164 PC12 cell proteins that have diverse molecular functions such as binding, catalysis, regulation, structural activity, etc., thereby extensively affecting the biological processes in the PC12 cells, particularly protein metabolism, response to stimulus, substance transport, and nucleic acid metabolism, with 56.71%, 42.07%, 29.88% and 28.66% of the identified proteins being involved in these biological processes, respectively. By interacting with the relevant proteins, LETX-VI enhanced the synthesis of dopamine; positively regulated cell division and proliferation; and negatively regulated cell cycle arrest, cell death, and apoptotic processes, and therefore has limited cytotoxicity against the PC12 cells, which were further experimentally confirmed. In general, the effects of LETX-VI on PC12 cells are more regulatory than cytotoxic. These findings have deepened our understanding of the action mechanism of LETX-VI on nerve cells and provided valuable clues for further related researches including those on Parkinson’s disease.

## 1. Introduction

Animal venoms contain various kinds of proteinaceous neurotoxins, which affect neurons in many aspects, such as specifically acting ion channel activities and influencing neurotransmitter release, and thus show valuable potentials in analysis of the structure-function of the nervous system and in the development of drugs for treatment of related diseases [1,2,3,4,5]. During the related research, exploring the mechanism of action of the neurotoxins is a fundamental and challenging work. There is a series of strategies for uncovering the action mechanism, such as patch clamp recording, receptor identification and the receptor-mediated signal transduction analysis, etc., [5,6,7,8]. Among other strategies, the pull-down assay is an important method for understanding the action mechanism of a toxin. The pull-down assay uses a purified and tagged proteinaceous toxin as a “bait” to bind its interaction proteins. When the method is used, the tagged toxin is first immobilized on an affinity ligand specific to the tag, producing an immobilized toxin to capture prey proteins, which can be from cell lysates, purified protein samples, etc. Such a method can be used to detect physical interactions between the toxin and the target proteins, confirm the predicted toxin interaction proteins, and understand the toxin-affected cellular processes [9,10,11]. Therefore, pull-down combined with mass spectrometry and bioinformatics can provide comprehensive information on the action mechanism of a proteinaceous neurotoxin and provide clues to guide further experimental researches, particularly suitable for the initial stage of a toxin research.

Latroeggtoxin-VI (LETX-VI) is a peptide neurotoxin with a molecular weight of 6196, mined from the transcriptome of spider *Latrodectus tredecimguttatus* eggs. Preliminary activity screen showed that LETX-VI inhibited Na^+^ channels in ND7/23 cells and promoted dopamine release from PC12 cells, without obvious toxicity against *Periplaneta americana* and bacteria as well as fungi including *Staphylococcus aureus*, *Candida albicans*, etc., demonstrating that LETX-VI is a mammal-specific neurotoxin [12,13]. Our present work was designed to probe into the molecular mechanism underlying the effects of LETX-VI on PC12 cells using a strategy based on the pull-down assay, so as to lay a foundation for the applications of LETX-VI in the research on nervous system and the development of drugs to treat related diseases.

## 2. Results

### 2.1. His-Tagged LETX-VI Preparation and Interaction Protein Identification

**Figure 1:**
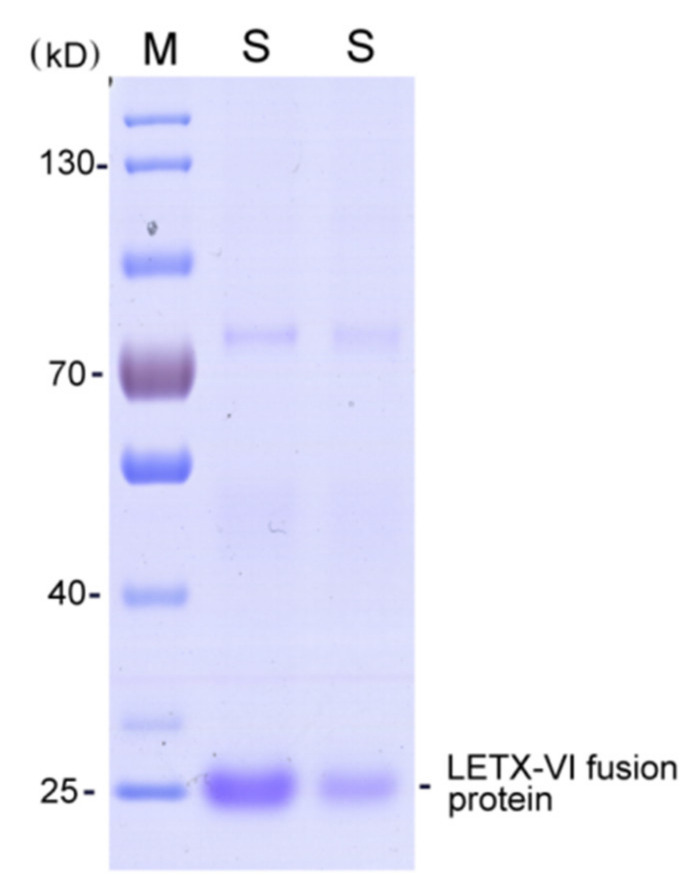
SDS-PAGE of the recombinant His-tagged LETX-VI affinity purified with Ni-NTA beads. M, molecular weight marker; S, affinity purified His-tagged LETX-VI fusion protein, with different loading amounts.

For the immobilization of LETX-VI acting as a “bait” protein, the LETX-VI was prepared in the form of His-tagged fusion protein using the expression vector pET32a and protein-producing strain *E. coli* BL21 (DE3). The affinity purified His-tagged LETX-VI (Figure 1) was then fixed on the Ni-NTA bead via the affinity between the His tag and the Ni-beads. After the PC12 cell lysate was precleared with Ni-NTA bead-bound His tag protein, the LETX-VI interaction proteins from the PC12 cells were captured and analyzed with a proteomic strategy. As a result, a total of 164 LETX-VI interaction proteins were identified (Supplementary Table S1). We systematically analyzed the main information on these proteins so as to get a comprehensive insight into the effects of LETX-VI on PC12 cells.

### 2.2. MW and pI Distributions

**Figure 2:**
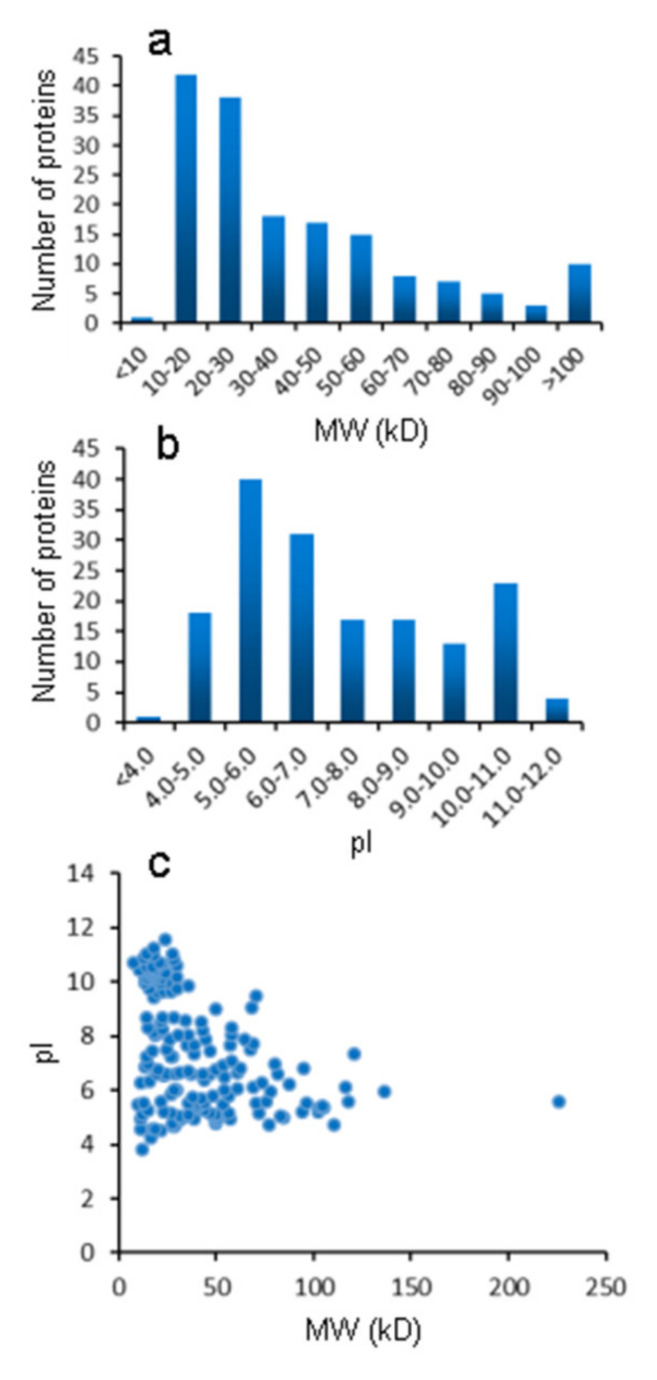
Analysis of the MW and pI distribution of LETX-VI interaction proteins. (**a**) MW distribution. (**b**) pI distribution. (**c**) Relationship between pI and MW of each identified proteins.

Statistical analysis showed that the MWs of the identified LETX-VI interaction proteins ranged from 7.8 to 226.2 kDa (Supplementary Table S1), and the MW distribution is shown in Figure 2a. Only one protein had a MW of smaller than 10 kDa; 80 proteins (accounting for 48.78% of the total identified proteins) were distributed in the MW range of 10–30 kDa; proteins with higher MW were relatively less. In the MW range above 10 kDa, the higher the MW, the fewer proteins were identified. The pI values of the identified proteins were distributed in the range of 3.84 to 11.55 (Supplementary Table S1). Comparatively, even more proteins (71, 43.29%) had their pI distributed in the range of 6.0 to 7.0. Note that a fairly high proportion of proteins (27, 16.46%) had a fairly high pI value (10.0–11.55) (Figure 2b). Figure 2c visually shows the relationship between pI and MW of each identified proteins. Most of the identified proteins have a MW smaller than 100 kDa and a distribution range from weak acidity to weak alkalinity, with a few proteins being distributed outside the MW and pI ranges. Relatively, LETX-VI bound even more low molecular weight proteins with a wide pI distribution range.

### 2.3. Subcellular Distribution

**Figure 3:**
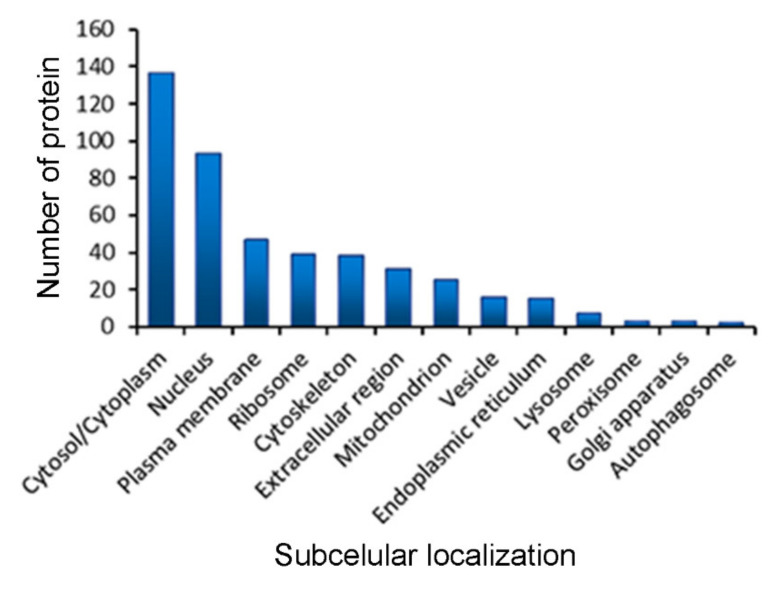
Subcellular localizations of LETX-VI interaction proteins.

GO information on the cell component indicated that each identified LETX-VI interaction proteins had one or more subcellular localizations (Supplementary Table S1). We analyzed the subcellular distributions of the identified proteins based on the 13 classifications: cytosol/cytoplasm, nucleus, plasma membrane, ribosome, cytoskeleton, extracellular region, mitochondrion, vesicle, endoplasmic reticulum, lysosome, peroxisome, Golgi apparatus, and autophagosome. The subcellular distribution profile of the identified proteins is shown in Figure 3. The cytosol/cytoplasm was the uppermost main subcellular compartment, with 137 cytosolic proteins (accounting for 83.54%) participating in the interaction with LETX-VI, followed by nucleus (93, 56.71%), plasma membrane (47, 28.66%), ribosome (39, 23.78%), cytoskeleton (38, 23.17%), etc. These data demonstrate that LETX-VI primarily interacts with the cytosolic and nucleic proteins, and some proteins in other subcellular organelles and even in the extracellular region may also become the interaction partners of LETX-VI.

### 2.4. Molecular Function

**Figure 4:**
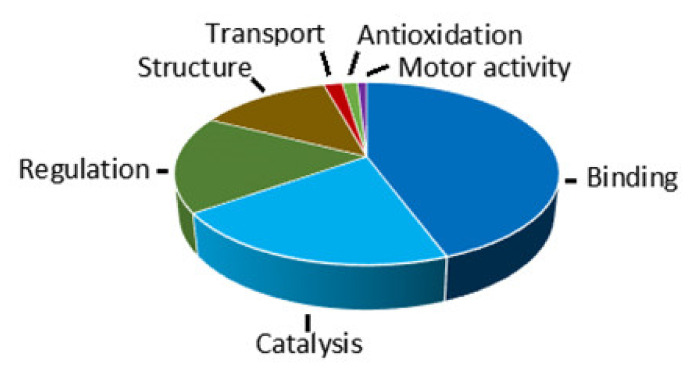
Molecular functions of LETX-VI interaction proteins.

The retrieved GO terms on the molecular function of the identified proteins indicated that a protein usually has more than one molecular function (Supplementary Table S1). We classified the molecular functions of the identified LETX-VI interaction proteins into seven groups: binding, catalysis, structure, regulation, transport, antioxidation, and motor activity (Figure 4). Figure 4 shows that the proteins with binding function constituted the largest group, which contained 144 proteins, accounting for 87.80% of the total identified LETX-VI interaction proteins. The LETX-VI-bound cellular components included proteins, enzymes, receptors, cytoskeleton, transcription factors, nucleic acids, nucleotides, phospholipids, inorganic ions, etc., suggesting that LETX-VI may affect multiple cellular processes via interacting with the proteins with binding functions. The second large group consisted of 68 proteins (accounting for 41.46%) with catalytic activity. These enzymes catalyze the reactions, particularly those involved in protein metabolism and its regulation in the PC12 cells, such as phenylalanine-tRNA ligase activity, protein-disulfide reductase activity, metalloendopeptidase activity, protein serine/threonine kinase activity, protein serine/threonine phosphatase activity, prostaglandin-E synthase activity, ATPase activity, etc.

More than one-third (34.15%) of the LETX-VI interaction proteins had regulatory activity, such as tyrosine 3-monooxygenase and L-dopa decarboxylase activator activity, translation regulator activity, ubiquitin ligase inhibitor activity, peptidase inhibitor activity involved in apoptotic process, DNA-binding transcription activator activity, DNA polymerase processivity factor activity, transcription coactivator activity, ribonuclease inhibitor activity, protein kinase regulator activity, protein phosphatase activator activity, phospholipase A2 inhibitor activity, ion channel inhibitor activity, transmembrane signaling receptor activity, etc. These proteins participated in the effects of LETX-VI on the metabolic processes in the PC12 cells mostly via modifying the activities of the relevant enzymes. Besides, the proteins having structural activity also constituted a fairly large group (43 proteins, accounting for 26.22%). Most of the proteins in this group were structural constituents of ribosome and cytoskeleton proteins such as tubulin; some of them were also involved in protein synthesis and substance transport. In addition, there were some LETX-VI interaction proteins having the activities of transport (6, 3.66%), antioxidation (5, 3.15%), and motor (3, 1.83%). Although these proteins only accounted for a small percentage, they were speculated to still play important roles in the effects of LETX-VI on the PC12 cells.

### 2.5. Biological Process

**Figure 5:**
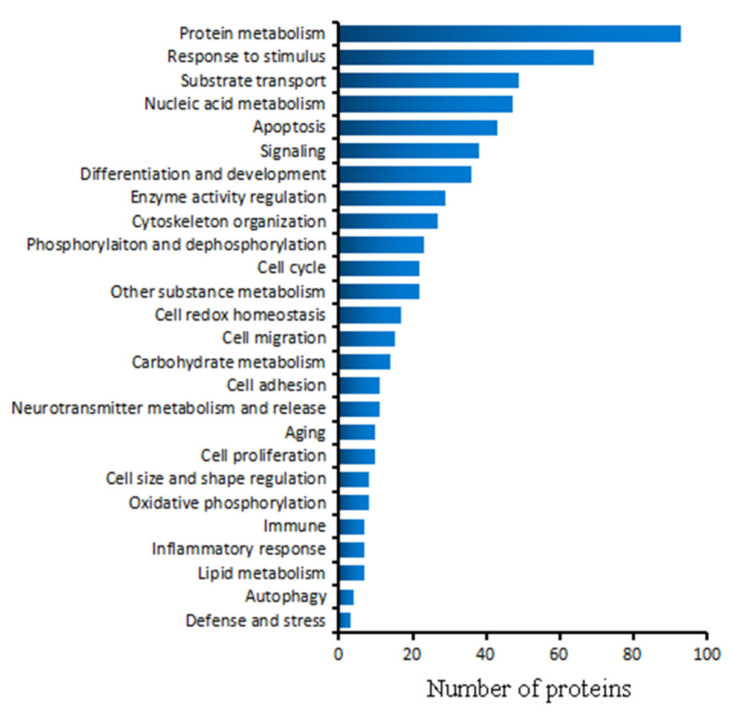
Biological processes of LETX-VI interaction proteins.

When we analyzed the biological processes that the identified LETX-VI interaction proteins were involved in, the retrieved GO terms on biological processes were grouped into 26 classifications according to the relativity of the terms (Figure 5 and Supplementary Table S1). Figure 5 shows that, of the 164 LETX-VI interaction proteins identified, 93 proteins were involved in protein metabolism, accounting for the largest proportion (56.71%). These proteins were involved in various aspects of protein metabolism, particularly protein synthesis, such as eukaryotic translation initiation factor 4H, elongation factor 1-alpha 1, and elongation factor 2 involved in initiation and elongation of protein synthesis; heat shock cognate 71 kDa protein, heat shock protein HSP 90-beta, and protein disulfide-isomerase associated with protein folding; and proteasome subunit alpha type-7, 26S proteasome non-ATPase regulatory subunit 1, and E3 ubiquitin-protein ligase NEDD4 related with proteasome-mediated protein catabolic process. Besides, a total of nearly 40 ribosomal proteins were identified to interact with LETX-VI. In contrast, the numbers of LETX-VI interaction proteins involved in the metabolisms of carbohydrates and lipids were much less, being only 14 and 7, respectively.

Sixty-nine proteins, constituting the second largest classification (42.07%), participated in the cellular response to various stimuli caused by for example endoplasmic reticulum stress, antigen, oxidative stress, hormone, drug, light, heat, etc., suggesting that LETX-VI may mediate the adaption of PC12 cells to the changes in the internal and external environment to a fairly large degree. The proteins involved in substance transport also accounted for a high percentage (29.88%). The transported substances included various cellular components such as biomacromolecules, low molecular weight organic substances and inorganic ions, etc., such as proteins, RNAs, cholesterol, and Ca^2+^. It was suggested that, by transporting these substances, LETX-VI affected the substance and energy metabolisms as well as their regulation. The proteins related to nucleic acid metabolism accounted for 28.66% of the total identified 164 proteins, including those involved in DNA replication, transcription, RNA processing, nucleotide metabolism, etc. In addition, the LETX-VI interaction proteins were also associated with a series of other biological processes, including apoptosis, signaling, differentiation and development, enzyme activity regulation, cell cycle, neurotransmitter metabolism and release, etc., although the numbers of relevant proteins were relatively less.

### 2.6. Effect of LETX-VI on Dopamine of PC12 Cells

**Figure 6:**
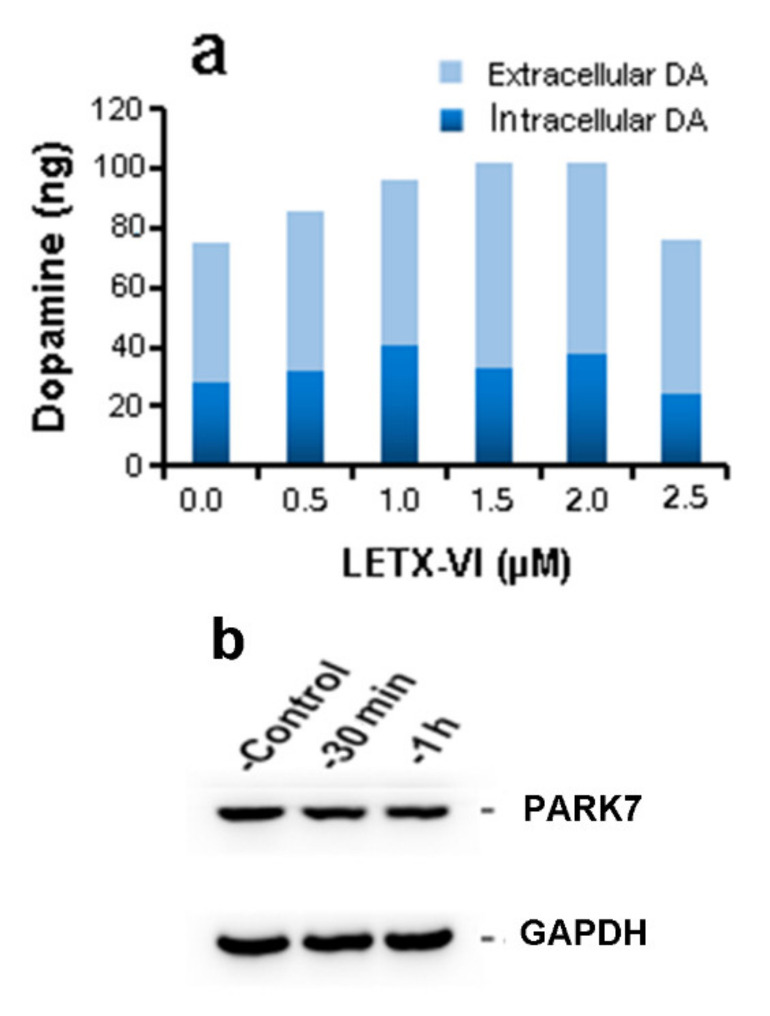
Effect of LETX-VI on dopamine (**a**) and Parkinson disease protein 7 (**b**) of PC12 cells. PARK7, Parkinson disease protein 7. GAPDH was used as a loading control.

The pull-down assay indicated that LETX-VI may interact with a homolog of Parkinson disease protein 7 (Parkinson disease protein 7 homolog) that has tyrosine 3-monooxygenase and L-dopa decarboxylase activator activity (Supplementary Table S1), suggesting that LETX-VI may promote the biosynthesis of dopamine in PC12 cells because tyrosine 3-monooxygenase (also named tyrosine hydroxylase) is the limited-rate enzyme of dopamine synthesis and L-dopa decarboxylase is also an important enzyme in the synthesis of dopamine (see Discussion). We experimentally assayed the extracellular (released) and intracellular dopamine contents after treatment of the cultured PC12 cells with LETX-VI at different concentrations (0, 0.5, 1.0, 1.5, 2.0, and 2.5 μM) for 20 min, respectively. The results in Figure 6a show that LETX-VI within the lower concentration range increased the amount of both extracellular and intracellular dopamine, and the total amount of dopamine of the PC12 cells was increased. These data demonstrated that the synthesis of dopamine in the PC12 cells was indeed enhanced, which is consistent with the pull-down assay. At higher concentration, the effect of LETX-VI on dopamine was not obvious, which may involve even more complex action mechanism. At the same time, we detected Parkinson disease protein 7 with blot analysis (Figure 6b). The result showed that PC12 cells expressed Parkinson disease protein 7 and treatment with LETX-VI at 1.5 μM for 30 and 60 min did not alter the level of this protein, suggesting that LETX-VI activated the activity of tyrosine 3-monooxygenase and L-dopa decarboxylase via interaction with Parkinson disease protein 7 homolog or Parkinson disease protein 7, not changing their expression levels.

### 2.7. Cytotoxicity of LETX-VI toward PC12 Cells

**Figure 7:**
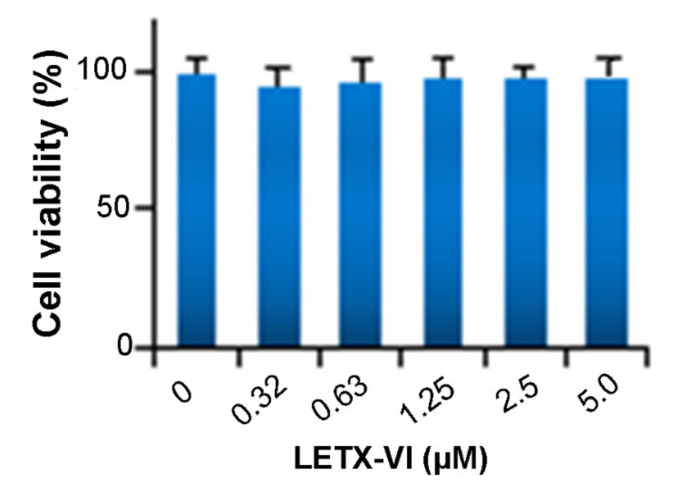
Effect of LETX-VI on viability of PC12 cells (*n* = 4). The PC12 cells were treated with LETX-VI for 4 h.

When we probed the effect of LETX-VI on PC12 cells via analyzing the LETX-VI interaction proteins, we found a batch of proteins that was associated with the viability, cell cycle, and apoptosis of PC12 cells. The GO information on their molecular functions and biological processes suggested that the cytotoxic effect of LETX-VI on PC12 cells should be low (see Discussion). In order to confirm the possible cytotoxicity of LETX-VI, we first detected the effect of LETX-VI on viability of PC12 cells. The resultant data (Figure 7) indicated that treatment of PC12 cells with LETX-VI up to 5.0 μM for 4 h did not significantly decrease the viability of the PC12 cells (*p* > 0.05).

**Figure 8:**
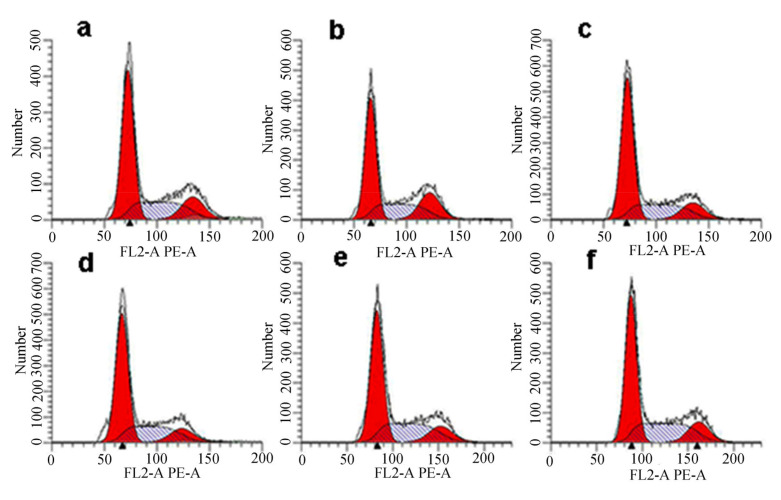
Effect of LETX-VI on cell cycle phase distribution of PC12 cells. (**a**) Control. (**b**–**f**) Treatment with LETX-VI at 0.32, 0.63, 1.25, 2.5, and 5.0 μM for 24 h, respectively.

For further investigation of the cytotoxicity of LETX-VI toward PC12 cells, we also detected the effect of LETX-VI on cell cycle phase distribution of PC12 cells. The results showed that, compared with those of the control, the percentages of G_0_/G_1_, S, and G_2_/M phases were not significantly changed after treatment with LETX-VI at different concentrations (0.32, 0.63, 1.25, 2.5, and 5.0 μM) for 24 h, although there were minor fluctuations. Figure 8 presents the representative histograms of flow cytometric analysis of cell cycle phase distribution in PC12 cells. In addition, there was no obvious sub-G_1_ peak, one of the apoptosis markers, before the G_0_/G_1_ peak, suggesting that the apoptosis caused by LETX-VI should be less.

**Figure 9:**
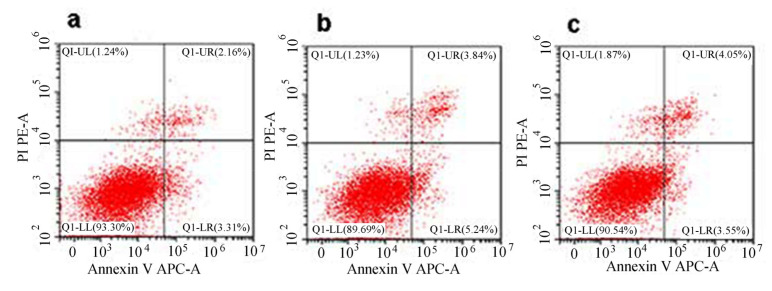
Effect of LETX-VI on apoptosis in PC12 cells. (**a**) Control. (**b**) 2.5 μM LETX-VI. (**c**) 5.0 μM LETX-VI. Treatment time was 24 h.

In order to confirm the effect of LETX-VI on apoptosis in PC12 cells, we made a flow cytometric analysis of apoptosis in PC12 cells after treatment with LETX-VI at 2.5 and 5.0 μM, two relatively higher LETX-VI concentrations that were used. The results indicated that LETX-VI at the test concentrations did not significantly alter the apoptosis rate as compared with the control, once again demonstrating that, as indicated by pull-down assay, the cytotoxicity of LETX-VI is relatively low. Figure 9 shows representative dot plot diagrams of flow cytometric analysis of PC12 cell apoptosis after LETX-VI treatment.

## 3. Discussion

After LETX-VI from the eggs of *L. tredecimguttatus* was found to be a neurotoxin [12], its effects on nerve cells and the underlying molecular mechanism aroused our attention. PC12 cells are derived from a pheochromocytoma of rat adrenal medulla and they can synthesize, store, and release catecholamines, mainly dopamine and norepinephrine, therefore becoming a commonly used neuron model to perform the relevant research [14,15,16,17,18]. In our present work, as an initiation step toward understanding the mechanism of action of LETX-VI on PC12 cells, the pull-down technique was employed, which, combining with mass spectrometry and bioinformatics, provided comprehensive information on the action mechanism of LETX-VI and guided the further experimental research. During the pull-down analysis, the immobilized LETX-VI was used as a “bait” protein. Before LETX-VI pull-down was performed, the PC12 cell lysate was precleared with Ni-NTA bead-bound His tag protein. Besides, incubation of the immobilized LETX-VI with the precleared lysate was followed by extensive washing. Therefore, the proteins pull-downed by the immobilized LETX-VI represented the LETX-VI-interacting partners. Mass spectrometry and protein database searching identified a total of 164 such proteins (Supplementary Table S1). In order to get a comprehensive insight into the effects of LETX-VI on the PC12 cells, we systematically analyzed the physiocochemical properties and GO annotations of the identified LETX-VI interaction proteins. Generally speaking, most of the identified proteins have a MW < 100 kDa, are distributed in a range of from weak acidity to weak alkalinity, and have a cytosol/cytoplasm subcellular localization. A finding worthy of noting is that a fairly high proportion of proteins (27 proteins, accounting for 16.46%) have a higher pI value (10.0–11.55) (Figure 2b,c). The interaction between these proteins and LETX-VI may involve some special molecular mechanism, which needs further investigation. The identified 164 LETX-VI interaction proteins have multiple molecular functions, including binding, catalysis, regulation, structural activity, etc., indicating that the effects of LETX-VI on PC12 cells are extensive. Particularly, 144 proteins (accounting for 87.80%) have binding function (Figure 4), suggesting that one of important action mechanisms of LETX-VI on PC12 cells is to “indirectly” affect multiple cellular processes through interacting with the proteins with binding function.

As LETX-VI was found to promote dopamine release from PC12 cells in previous work [12], we paid special attention to the identified proteins with functions closely related to dopamine. GO annotations indicated that the identified protein Parkinson disease protein 7 homolog has similar function as Parkinson disease protein 7 (alternative name: protein DJ-1) that had been proven to have tyrosine 3-monooxygenase (also known as tyrosine hydroxylase, TH) and L-dopa decarboxylase (DDC) activator activity [19]. Besides, Parkinson disease protein 7 transcriptionally upregulates the TH. Inactivation of Parkinson disease protein 7 by small interference RNA (siRNA) results in a decreased TH expression and DDC production in human dopaminergic cell lines [20]. TH is the rate-limiting enzyme in the synthesis of dopamine, catalyzing tyrosine into L-dopa, which is decarboxylated by DDC into dopamine [14,21]. Parkinson disease is caused by loss of dopamine, which is synthesized from tyrosine by TH and DDC. Parkinson disease protein 7 directly bound to the two enzymes to positively regulate their activities. Loss-of-function mutations in Parkinson disease protein 7 cause a subset of familial Parkinson’s disease [19,20]. These data suggest that LETX-VI might enhance the biosynthesis of dopamine via interacting with Parkinson disease protein 7 homolog or Parkinson disease protein 7. In addition, several other LETX-VI interaction proteins were also closely related to the transport and release of neurotransmitters, such as heat shock cognate 71 kDa protein involved in synaptic vesicle uncoating and vesicle-mediated transport, annexin A1 positively regulating vesicle fusion, etc. These findings provide a partial explanation for the mechanism underlying the promoting effect of LETX-VI on the release of dopamine from PC12 cells [12]. Furthermore, LETX-VI is thus suggested to have potentials in the relevant pathological study and the treatment of Parkinson’s disease. In order to confirm the effect of LETX-VI on dopamine, we experimentally measured the extracellular (released) and intracellular dopamine of the cultured PC12 cells treated with LETX-VI. The results demonstrated that LETX-VI treatment led to an increase in total dopamine, and LETX-VI was able to promote dopamine release from the PC12 cells (Figure 6), which supports the results of the pull-down assay and demonstrated the guidance action of the pull-down assay in the related researches.

Ascertaining the PC12 cellular processes affected by LETX-VI and the differences in the amplitude of influence is greatly helpful to understand the effects of LETX-VI on the PC12 cells. Therefore, we made a detailed analysis on the biological processes that the LETX-VI interaction proteins were involved in. The results demonstrated that more than half of the LETX-VI interaction proteins (56.71%) were involved in protein metabolism, particularly protein synthesis, including more than 40 ribosomal proteins and relevant regulatory proteins (Supplementary Table S1), whereas the proteins involved in the metabolisms of carbohydrates and lipids were much less (Figure 5). These results indicated that the effect of LETX-VI on protein metabolism was stronger than that on carbohydrate and lipid metabolisms with respect to the numbers of identified relevant proteins. Likewise, in terms of the number of identified LETX-VI interaction proteins, the effect of LETX-VI on nucleic acid metabolism was weaker than that on protein metabolism but much stronger than those on carbohydrate and lipid metabolisms. These findings suggest that LETX-VI affects protein expression in PC12 cells not only at translation level, but also at DNA replication and transcription levels, although the affecting degree is different. Besides protein metabolism, the cellular response to various stimuli and substance transport were also the cellular processes that were heavily affected by LETX-VI as the numbers of the identified LETX-VI interaction proteins involved in them were only secondary to that involved in protein metabolism.

In addition, the LETX-VI interaction proteins are also associated with a series of other biological processes, including apoptosis, signaling, differentiation and development, enzyme activity regulation, etc. Although the numbers of the proteins involved in these processes are relatively less, their roles in the effects of LETX-VI on PC12 cells are not negligible. For getting an insight into the possible cytotoxic effect of LETX-VI on PC12 cells, we deliberately analyzed the identified proteins that were associated with the proliferation, cell cycle, and apoptosis of PC12 cells. A series of related proteins were found. For example, heat shock protein beta-1 positively regulated cell division and cell population proliferation, and negatively regulated apoptotic signaling pathway and apoptotic process; heat shock protein HSP 90-beta negatively regulated cell cycle arrest and neuron apoptotic process; and Parkinson disease protein 7 homolog and heat shock 70 kDa protein 4 negatively regulated the apoptotic process and cell death. Although there were a few proteins having the opposite function (Supplementary Table S1), the specific regulatory functions of most related proteins suggest that the cytotoxic effect of LETX-VI on PC12 cells, if present, is low. Based on the clues, we designed experiments and detected the effects of LETX-VI on the viability, cell cycle, and apoptosis of PC12 cells. The resultant data showed that exposure of PC12 cells to LETX-VI up to 5.0 μM for 4 h did not obviously decrease the viability of the PC12 cells (Figure 7) and LETX-VI treatment for 24 h led to no significant alternations in the cell cycle phase distribution and apoptosis rate of the cells (Figure 8 and Figure 9), which were consistent with results of the pull-down assay.

## 4. Conclusions

**Figure 10:**
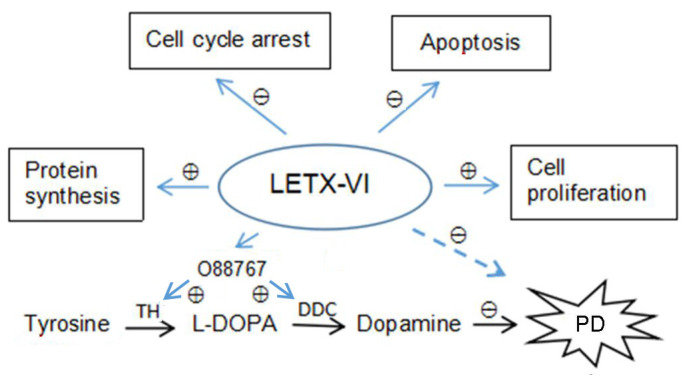
Selected significant regulatory effects of LETX-VI on the biological processes in PC12 cells as well as the implications in Parkinson’s disease. LETX-VI: latroeggtoxin-VI. 088767: Parkinson disease protein 7 homolog accession. TH: tyrosine hydroxylase. DDC: L-dopa decarboxylase. PD: Parkinson’s disease. ⊕: positive regulation. ⊖: negative regulation.

Using a pull-down assay-based strategy, LETX-VI was shown to interact with a series of PC12 cell proteins that have diverse molecular functions such as binding, catalysis, regulation, structural activity, etc., thereby extensively affecting the biological processes in the PC12 cells particularly protein metabolism, response to stimulus, substance transport, nucleic acid metabolism, and so on. By interacting with the relevant proteins, LETX-VI enhanced the synthesis of dopamine, positively regulated cell division and proliferation, and negatively regulated cell cycle arrest, cell death, and apoptotic process, and thus was lowly cytotoxic toward the PC12 cells, which was further experimentally confirmed. Generally speaking, the effects of LETX-VI on PC12 cells are more regulatory than cytotoxic, and LETX-VI is suggested to have potentials in the relevant pathological study and the treatment of Parkinson’s disease. This work has not only deepened our understanding of the effects of LETX-VI on nerve cells and supplied valuable clues for further related researches including those on Parkinson’s disease (Figure 10), but also provided a referential research strategy.

## 5. Materials and Methods

### 5.1. Reagents

Dulbecco’s modified Eagle’s medium (DMEM) and fetal bovine serum (FBS) were purchased from Gibco BRL (Grand Island, NY, USA). Dithiothreitol (DTT), iodoacetamide (IAA), sequencing grade trypsin, and protease inhibitor cocktail were from Sigma-Aldrich (St. Louis, MO, USA). Trihydroxymethy aminomethane (Tris) and enterokinase were from Sangon Biotech (Shanghai, China). Iodine was from Thermo Scientific (Waltham, MA, USA). Cell counting kit-8 (CCK-8) was from Beyotime Biotechnology (Shanghai, China). All other reagents used were of the highest grade available.

### 5.2. Preparation of His-Tagged LETX-VI

Recombinant LETX-VI fusion protein was prepared through heterologous expression in *E. coli* based on the method described [12]. Briefly, the total RNA extracted from *L. tredecimguttatus* eggs was used as the template for synthesis of the first cDNA strand. Nest PCR was employed to amplify the LETX-VI gene, which was cloned into expression vector pET32a. Expression of His-tagged LEX-VI fusion protein was carried out using *E. coli* BL21 (DE3) by IPTG induction. The expressed fusion protein was affinity purified with Ni-NTA beads and analyzed with SDS-PAGE.

### 5.3. PC12 Cell Culture and HIS Pull-Down

PC12 cells purchased from Shanghai Life Science Institute (Shanghai, China) were incubated in 6 cm culture dishes containing DMEM supplemented with 10% FBS. The culture dishes were placed in a 37 °C incubator containing humidified air (95%) and carbon dioxide (5%) until the PC12 cells were grown to reach approximately 90% confluence. For the pull-down assay, the culture medium was sucked away and the PC12 cells were washed twice with PBS, followed by cell collection and homogenization in a buffer (83 mM sucrose, 6.6 mM 1,3-diazole, protease and phosphatase inhibitor cocktail, pH 7.0). The supernatant was recovered after centrifugation at 14,000× *g* for 15 min at 4 °C, and then precleared by incubation with Ni-NTA bead-bound HIS tag protein overnight at 4 °C under slow continuous rotation. The precleared cell lysate was incubated with Ni-NTA bead-bound His-tagged LETX-VI fusion proteins overnight at 4 °C under slow continuous rotation. Following the incubation, the beads were collected by centrifugation and washed with the buffers (20 mM 1,3-diazole, 20 mM Tris-Cl, pH 8.0) containing 150 mM and 300 mM NaCl sequentially. The bound proteins were eluted from the beads with an elution buffer (300 mM NaCl, 500 mM 1,3-diazole, 20 mM Tris-Cl, pH 8.0).

### 5.4. In-Solution Digestion

For in-solution digestion, 180 µg of LETX-VI interaction proteins was dissolved in 200 µL of 0.1 mM Tris-HCl buffer and boiled for 3 min. The proteins in the solution were reduced with 10 mM DTT at 37 °C for 2 h and alkylated by 50 mM IAA in the dark at room temperature for 30 min. After the mixture was diluted to 1 mL with the 0.1 mM Tris-HCl buffer, trypsin was added at an enzyme to protein ratio of 1:50 (*w*/*w*) and incubated overnight at 37 °C. The enzymatic digestion was stopped by addition of 0.1% formic acid and the mixture was lyophilized for CapLC-MS/MS analysis.

### 5.5. CapLC-MS/MS Analysis and Bioinfomatics

The CapLC-MS/MS analysis of the tryptic peptides was performed on an Easy-nLC 1200 HPLC system (Thermo Scientic, MA, USA) coupled with an Orbitrap Exploris 480 mass spectrometer (Thermo Scientic, MA, USA). Buffer A was 0.1% formic acid and buffer B was ACN containing 0.1% formic acid. The peptides were eluted from the nano-scale analytical column (PepMap C18, 75 μm × 25 cm) with a gradient from 5% to 30% buffer B over 70 min at a flow rate of 200 nL/min. The column temperature was set at 55 °C. The eluted peptides were directed into the ion source of the mass spectrometer for MS and MS/MS analyses. The main parameters were set as follows: electrospray voltage 2.3 kV, ion source temperature 320 °C, Full MS scan range m/z 350–1600 with a resolution 120,000 at m/z 200. The 20 most abundant ions meeting the requirements for MS/MS fragmentation in each MS scan were selected for MS/MS fragmentation by higher energy C-trap dissociation (HCD) and MS/MS scan was performed at a resolution of 17,500.

The acquired raw spectral data were processed with the Proteome Discoverer software and the Sequest algorithm was employed to search protein databases for protein identification. Main search parameters were set as follows: database, Swissport (https://www.uniprot.org (accessed on 29 January 2021)); enzyme, trypsin; allowance of up to two missed cleavage sites; MS mass tolerance, 10 ppm; MS/MS mass tolerance, 0.02 Da; fixed modification, carbamidomethylation (C); variable modification, oxidation (M), deamidation (N). After database searching, the peptide identifications were validated using the PeptideProphet algorithm; only those with high confidence were accepted. The false discovery rate (FDR) was set to 0.01. The main physiocochemical parameters and gene ontology (GO) annotations of the identified proteins were retrieved from the UniProt databases (https://www.uniprot.org (accessed on 29 January 2021)) and the linked database QuickGO (https://www.ebi.ac.uk/QuickGO (accessed on 29 January 2021)).

### 5.6. Detection of Effect of LETX-VI on Dopamine of PC12 Cells

PC12 cells, obtained from Shanghai Life Science Institute in Shanghai of China, were cultured according to method described [2]. Briefly, the PC12 cell were incubated in culture dishes containing DMEM supplemented with 10% FBS in an incubator at 37 °C in a humidified atmosphere of 5% CO_2_ and 95% air until the cells were grown to about 90% confluence. Then, the cells were divided into a 96-well to be subcultured to reach 80–90% confluence. PC12 cells in the wells were separately treated with LETX-VI at concentrations of 0 (control), 0.5, 1.0, 1.5, 2.0, and 2.5 μM for 20 min in serum-free culture medium. The cells and culture medium were separated by low speed centrifugation and the dopamine in the interior of cells and the culture medium was separately determined with a fluorescence spectrophotometer (Model F97, Lengguang Tech, Shanghai, China) using the method previously described [22].

### 5.7. Evaluation of Cytotoxicity of LETX-VI toward PC12 Cells

Effect of LETX-VI on PC12 cell viability was assessed using a Cell counting Kit-8 (CCK-8 kit) assay according to the instructions of manufacturer. PC12 cells were cultured an incubator at 37 °C in a humidified atmosphere of 5% CO_2_ and 95% air to about 90% confluence in DMEM containing 10%FBS. After being subcultured in a 96-well plate, the cultured PC12 cells were separately co-incubated with different concentrations of LETX-VI (0, 0.32, 0.63, 1.25, 2.5, 5.0 µM) for 4 h, followed by CCK-8 solution addition and OD_450nm_ measurement.

The flow cytometric assay was employed to evaluate the effects of LETX-VI on cell cycle and apoptosis in PC12 cells based on the method previously described [23]. The subcultured PC12 cells were exposed to LETX-VI at different concentrations (0, 0.32, 0.63, 1.25, 2.5, and 5.0 µM) in a humidified atmosphere of 5% CO_2_ and 95% air in an incubator at 37 °C for 24 h. Then, the cell cycle phase distribution of PC12 cells was measured. The apoptosis in the PC12 cells treated with 2.5 and 5.0 µM LETX-VI was also detected using the flow cytometric method.

## Data Availability

The data poduced in this study are available in the present article and Supplementary Table S1.

## Supplementary Materials

The following are available online at https://www.mdpi.com/2072-6651/13/2/136/s1, Supplementary Table S1. Main information on the identified LETX-VI interaction proteins.

## Abbreviations

CapLC-MS/MS	capillary liquid chromatography-tandem mass spectrometry
DDC	L-dopa decarboxylase
GO	gene ontology
LETX-VI	Latroeggtoxin-VI
MW	molecular weight
pI	isoelectric point
SDS-PAGE	sodium dodecylsulfate-polyacrylamide gel electrophoresis
TH	tyrosine hydroxylase
PD	Parkinson’s disease
